# Quality of Life of People Living with HIV/AIDS: A Cross-Sectional Study in Zhejiang Province, China

**DOI:** 10.1371/journal.pone.0135705

**Published:** 2015-08-26

**Authors:** Ma Liping, Xu Peng, Lin Haijiang, Ju Lahong, Lv Fan

**Affiliations:** 1 National Centre for AIDS/STD Control and Prevention, Chinese Centre for Disease Control and Prevention, Beijing, China; 2 Taizhou Centre for Disease Control and Prevention, Zhejiang province, China; National Center for AIDS/STD Control and Prevention, China CDC, CHINA

## Abstract

Health-related quality of life (HRQOL) has become a concept commonly used in the related research. Using the World Health Organization Quality of Life Questionnaire for Brief Version (WHOQOL-BREF), this study evaluated the Quality of Life (QOL) of people living with HIV/AIDS (PLWHA) in Zhejiang province, China, and assessed the influences of demographic, laboratory and disease-related variables on QOL. This cross-sectional study was conducted among PLWHA aged ≥ 18 years in Taizhou municipality, Zhejiang province, China, between August 1 and October 31, 2014. A multiple linear regression model was used to analyze the influential factors. Of 403 subjects, 72.48% were male, 72.46% had received a high- school or above education, 94.79% were of Han ethnicity, and 65.51% were non farmers. The total score of QOL was 15.99±1.99. The scores of QOL in physiological, psychological, social relation, and environmental domains were 14.99 ±2.25, 14.25 ±2.12, 13.22 ±2.37, and 13.31 ±1.99 respectively. Except the total score of QOL and the score of environmental domain (p<0.05), the scores in other domains had no significant difference with the results of the national norm level. The multiple linear regression model identified the physical domain related factors to be age (*β* = -0.045), CD4 count (*β* = 0.002), and ART adherence(*β* = 1.231). And it also showed that psychological domain related factors included CD4 count (*β* = 0.002) and WHO clinical stage (*β* = -0.437); social domain related factors included WHO clinical stage (*β* = -0.704) and ART adherence (*β* = 1.177); while environmental domain related factors included WHO clinical stage (β = -0.538), educational status(*β* = 0.549) and ART adherence(*β* = 1.078).Those who are young, with higher level of education, higher CD4 count and good access and adherence of ART, are likely to have better QOL among PLWHA in Zhejiang province. This suggests that in addition to ART, many other factors should be taken into consideration to improve the QOL of PLWHA. The relatively lower scores the subjects received in social relation and environmental domains also suggest that social relation and environmental interventions need to be strengthened.

## Introduction

Human immunodeficiency virus (HIV) / Acquired immune deficiency syndrome (AIDS) is a chronic infection that affects not only the patients’ physical condition, but also their social relations, mental health and financial aspects[1]. Since its start in 1981, AIDS has become a major health problem worldwide. There have been more than 78 million people infected with HIV by the end of 2013 with people living with HIV/AIDS amounted to 35 million[2]. Epidemic estimates show that China’s population of people living with HIV/AIDS (PLWHA) is about 780,000. Case reports of these patients reveal that 46.5% and 13.7% were infected through heterosexual transmission and homosexual transmission respectively. The proportion of the cases resulting from sexual transmission has increased from 33.1% in 2006 to 76.3% in 2011[3]. Sexual transmission has become the most prevalent transmission route, with sexual transmission between men increasing markedly[4]. Located in East China, Zhejiang is a coastal province with relatively developed economy and cultural values of openness. In Zhejiang, the majority of patients acquired HIV through sexual transmission, and the reported HIV infections have reached 11,357 by the end of 2012. The HIV prevalence rate among the general population is about 0.01% [5], far less than that in Henan and Yunnan province. In spite of its moderate HIV epidemic, risk factors such as high prevalence of sexually transmitted infections and various entertainment venues providing sex service in main urban areas are driving the epidemic in Zhejiang province [6]. The local municipality has attached great importance to improving the living and traffic environment of PLWHA.

In China, the National Free Antiretroviral Treatment Program (NFATP) initiated in 2002 has significantly reduced the mortality of HIV patients, from 27 deaths per 100 person-years prior to treatment to 4–5 after 6 months of ART combination therapy [7]. However, given the need of managing HIV/AIDS as a chronic and survivable disease and the medication side effects, opportunistic infections, and the constant stigma and discrimination experienced by the PLWHA, there has been growing concern about PLWHA’s overall wellbeing in physical, psychological and socioeconomic domains [8]. In China, the HIV/AIDS epidemic has evoked widespread discrimination and prejudice towards PLWHA. Like other countries, China is faced with the challenges of both controlling the epidemic and eliminating discrimination[9].The QOL of individuals living chronically with HIV and AIDS has been considered as one of the main treatment outcomes [10].

The term health-related quality of life (HRQOL) is often used to indicate QOL as it relates to diseases or treatments [11]. Quality of life (QOL) is used as an important outcome indicator for healthcare decision-making and intervention effects evaluation [12]. QOL can be defined as a subjective multidimensional evaluation of one’s functioning and well-being in day-to-day life [13]. Changes in HRQOL, including functional status, perceptions of other people, social opportunities, treatment requirements and disability, may last throughout the rest of the PLWHA’s life[14].

The WHOQOL is a cross-cultural instrument initiated and developed by the World Health Organization, and its brief version is to accelerate HRQOL assessment among populations worldwide. The WHOQOL-BREF has been recommended as the most suitable instrument for assessing the QOL of various people [13, 15, 16]. The WHOQOL-BREF has been translated into different languages, including the traditional Chinese, The Chinese version of WHOQOL-BREF, compared with WHOQOL-100, has been commended as good, valid and reliable with the high correlation in relevant domains. (Pearson correlation coefficients: social relation domain = 0.89, and physiological domain = 0.95) [17].

There have already been several studies on the assessment of QOL of PLWHA in China. [18–21]. However, this study was to assess QOL of PLWHA in Zhejiang province, where the main transmission route of HIV/AIDS is sexual transmission, and explore the associated factors affecting the QOL. As some other studies revealed, socio-demographic characteristics (age, gender, ethnicity, education level, marital status, employment, and transmission route) are the primary factors that affect the QOL of PLWHA. In this study, apart from these factors, the access and the adherence to ART were also included to clarify the associated factors that may impact on the QOL of participants.

## Methods

### Ethical considerations

This study was approved by the Institutional Review Board of National Center for AIDS/STD Control and Prevention, Chinese Center for Disease Prevention and Control on Jan 21 2014. Written informed consent was obtained from each participant. Participants were informed that participation in this study was purely voluntary and could be cancelled at any time; all information gathered in this study would be treated as confidential; all information about participants could only be accessed by the research team; and the contact information of participants would be deleted after the completion of this study.

### Study design

This is a cross-sectional study based on quantitative analysis. It was carried out in a local CDC and designated clinical hospitals specialized in supporting PLWHA in Taizhou, Zhejiang province, China from August 1 to October 31, 2014.

### Recruitment

The subjects of this study were recruited among the PLWHA in Taizhou. Taizhou is a HIV/AIDS treatment site of Zhejiang Province. The target subjects of this study need to meet the following criteria: (1) a confirmed diagnosis of HIV infection (2) 18 years older (3) mentally competent to answer questions. (4) Accessible for this survey. Following China’s national standards for ART management, Taizhou HIV/AIDS treatment site provides the ART combination therapy (two NRTIs plus one NNRTI) for those PLWHA eligible for ART. Patients on ART receive free laboratory test for CD4+ T cell counts once every three to six months and HIV viral load test once a year so as to evaluate the effect of the treatment. Once they have side effects or other health problems, they may go to the clinic and get a laboratory test. In Taizhou, by the end of October, 2014, 2479 patients had received free ART since the free ART programme was initiated in 2004.

### Measures

The socio-demographic and clinical information of subjects was collected directly from the case reporting database of HIV/AIDS Comprehensive Response Information Management System in Taizhou by the end of 2014, including characteristics such as age, gender, marital status, educational level, route of transmission, status of ART treatment, adherence of HAART, level of CD4 cell count, and WHO clinical stage, and so on.

The WHOQOL-BREF is a 26-item multiple-choice questionnaire, including one item for general QOL, one item for overall HRQOL and 24 items covering four domains (physical, psychological, social and environmental). The physical domain includes three facets: pain and discomfort, energy and fatigue, and sleep and rest. The psychological domain includes five facets: positive feelings, negative feelings, learning and concentration, bodily image, and self-esteem. The social domain includes three facets: personal relationships, practical social support, and sexual activity. The environmental domain includes five facets: financial resources, healthcare availability, opportunities for acquiring new information and skills, opportunities for leisure, and transport. Each facet consists of two to eight items. The equation recommended by WHO (World Health Organization, WHOQOL User Manua1, WHO, Geneva. 1998) is used to calculate the total score of each domain. Because the number of items varies across the four domains, the score of each domain is calculated by multiplying the average score of all items in the domain by the same factor of 4. With each item's score ranging from 1 to 5, the total score of each domain will be between 4 and 20. The higher the score, the better the QOL, which makes it convenient to compare the scores in different domains, population and studies. Besides, the WHOQOL-BREF is a self-administered questionnaire and gives the evaluation of the subject’s QOL in the recent four weeks.

The QOL of subjects was measured by Chinese version of the WHOQOL-BREF [17].The reasons why we chose the WHOQOL-BREF are as follows: (1) it has been widely used and validated in China[17], (2) it provides a comparison of HRQOL between different diseases [13, 22, 23], (3)no subject will have any difficulty in completing the questionnaire,(4) it has a certain degree of cultural compatibility, which makes the comparison between people from different countries possible.

### Statistical analysis

SPSS 18.0 (SPSS Inc., Chicago, IL, USA) was used for data analysis. Mean, standard deviation and percentage were used to describe data. U- test was applied to compare the result with the national Norm among China’s general population[24]. T-test was used to assess differences between the means of individual variables and differences in the mean scores of various domains of the WHOQOL- BREF. Differences between ≥3 groups were analyzed by the analysis of variance (ANOVA). Chi-squared tests or Fisher’s Exact tests were used to compare categorical variables. A multivariate regression model was developed, with social demographic, CD4 count, and medical history as independent variables and the QOL scores of the four domains as dependent variables, to assess the adjusted relationship between the QOL and the socio-demographic and clinical factors. Covariates with a p-value, 0.10 from the univariate analysis were entered into a full multivariate regression model and stepwise selection was used to include significant covariates. All hypothesis testing was based on 2-sided tests with an alpha level of 0.05. The continuous variables were entered into the multivariate regression directly, for instance age and CD4 count. However the ordinal or categorical variables were grouped and assigned with number, for example WHO clinical stage (1 = Ⅰstage,2 = Ⅱstage,3 = Ⅲ and Ⅳ stage), educational status (1 = None, 2 = Primary, 3 = Secondary, 4 = Tertiary and above), Marital status(1 = Single, 2 = Divorced or widowed, 3 = Married) and adherence of HAART(1 = leakage, 2 = no leakage).

## Results

### Social-demographic Characteristics of the Subjects

Between August 1 and October 31, 2014, 403 HIV-positive people completed the WHOQOL-BREF. Of them, 175 (43.43%) were hetero male; 117(29.05%) were homo male; and 111(27.05%) were female. The homo male (35.82±11.12) and female (37.98±11.33) groups tended to be younger than the hetero male (43.15±12.44). The subjects age ranged from 18 to 79, with the age group of 30 to 50 accounting for 60.3% .382(94.79%) were ethnically Han, and the rest were ethnically minority; 292(72.46%) had received the secondary or above education, and esp., 63(53.85%) homo men had got Tertiary or above education; 290(71.96%) had marital experience,228 (56.58%) were married at the time of data collection, and esp., the female were more likely to be married (hetero men 64.57% vs. homo men29.91% vs. the female 72.07%); not farmers were more than farmers (65.51% vs.34.49%), and difference among three groups of people are significant (*P* = 0.001), 77.92% were local residents, 22.08% were from outside, difference among three groups of people are significant (*P*<0.001), and esp., homo men and the female were more likely to come from outside; 68.49% were infected through heterosexual transmission while 29.03% through homosexual transmission, with significant difference between men and women (*P* = 0.001). The detailed socio-demographic and clinical characteristics of the subjects are presented in Table 1.

**Table 1:** Socio-demographic and clinical characteristics of the PLWHA on survey.

Characteristics	Male hetero (%)	Male homo(%)	Female (%)	Total (%)	x^2^	*P* value
**Gender**	175(43.43)	117(29.05)	111(27.55)	403(100.00)		
**Average age±SD (yrs)**	43.15±12.44	35.82±11.12	37.98±11.33	39.59±12.17		**>0.05**
**Age(yrs)**					**37.45**	**<0.001**
<30	16(9.14)	37(31.62)	26(23.42)	79(19.60)		
30∼	60(34.29)	38(32.48)	46(41.44)	144(35.73)		
40∼	52(29.71)	28(23.93)	19(17.12)	99(24.57)		
50∼	26(14.86)	10(8.55)	16(14.41)	52(12.90)		
≥60	21(12.00)	4(3.42)	4(3.60)	29(7.20)		
**ethnic**					**17.93**	**<0.001**
Han	169(96.57)	116(99.15)	97(87.39)	382(94.79)		
Minority	6(3.43)	1(0.85)	14(12.61)	21(5.21)		
**Educational status**					**70.30**	**<0.001**
None	8(4.57)	1(0.85)	11(9.91)	20(4.96)		
Primary	44(25.14)	13(11.11)	34(30.63)	91(22.58)		
Secondary	89(50.86)	40(34.19)	54(48.65)	183(45.41)		
Tertiary and above	34(19.43)	63(53.85)	12(10.81)	109(27.05)		
**Marital status**					**62.2**	**<0.001**
Single	39(22.29)	62(52.99)	12(10.81)	113(28.04)		
Married	113(64.57)	35(29.91)	80(72.07)	228(56.58)		
Divorced or widowed	23(13.14)	20(17.09)	19(17.12)	62(15.38)		
**Occupation**					**19.05**	**<0.001**
Farmer	81(46.29)	30(25.64)	28(25.23)	139(34.49)		
Not farmer	94(53.71)	87(74.36)	83(74.77)	264(65.51)		
**Area source**					**17.89**	**<0.001**
inner province	152(86.86)	89(76.07)	73(65.77)	314(77.92)		
other province	23(13.14)	28(23.93)	38(34.23)	89(22.08)		
**Transmission routes**					**403.47**	**<0.001**
heterosexual transmission	168(96.00)	0(0.00)	108(97.30)	276(68.49)		
homosexual transmission	0(0.00)	117(100.00)	0(0.0)	117(29.03)		
other transmission	7(4.00)	0(0.00)	3(2.70)	10(2.48)		
**Reported STD symptom(n = 396)**					**19.53**	**0.001**
Yes	31(18.13)	32(27.59)	12(11.01)	75(18.94)		
No	97(56.73)	69(59.48)	82(75.23)	248(62.63)		
NA	43(25.15)	15(12.93)	15(13.76)	73(18.43)		
**CD4 cell count(cells/mm** ^**3**^ **) (n = 397)**					**13.95**	**0.03**
≤200	38(22.22)	14(11.97)	12(11.01)	64(16.12)		
201∼349	43(25.15)	36(30.77)	26(23.85)	105(26.45)		
350∼499	52(30.41)	28(23.93)	38(34.86)	118(29.72)		
≥500	38(22.22)	39(33.33)	33(30.28)	110(27.71)		
**On HAART**					**2.20**	**0.333**
Yes	126(72.9)	87(74.36)	73(65.77)	286(70.97)		
No	49(28.00)	30(25.64)	38(34.23)	117(29.03)		
**WHO clinical stage(n = 285 on HAART)**					**10.88**	**0.028**
Ⅰ stage	60(48.00)	60(68.97)	41(56.16)	161(56.49)		
Ⅱ stage	45(36.00)	19(21.84)	26(35.62)	90(31.58)		
Ⅲ and Ⅳ stage	20(16.00)	8(9.20)	6(8.22)	34(11.93)		
**Adherence of HAART(n = 286)**					2.64	0.267
Leakage of drug	115(91.27)	80(91.95)	62(84.93)	257(89.86)		
No leakage of drug	11(8.73)	7(8.05)	11(15.07)	29(10.14)		
**Treatment duration(n = 285 on HAART)**					**11.35**	**0.023**
≤24 months	61(48.80)	46(52.87)	24(32.88)	131(45.96)		
25~48 months	35(28.00)	28(32.18)	23(31.51)	86(30.18)		
≥49 months	29(23.20)	13(14.94)	26(35.62)	68(23.86)		

Key: HIV–Human immunodeficiency virus, SD–standard deviation, HAART–highly active antiretroviral therapy

### Medical history

Table 1 shows that 62.63% of subjects had no reported STD symptom in the past, while 18.94% did have, and, esp., 27.59% of homo men had ever reported their STD symptoms; 286 (70.97%) were on ART and the median duration of treatment was 31.54 months (range: 3–106 months, 45.96% for less than 2 year, 30.18% for more than 2 but less than 4 years, 23.86% for more than 4 years).The table also reveals that compared with men, women were more likely to adhere to the long-term ART treatment, but the gender difference wasn’t significant. There were no obvious differences in levels of CD4 cell count and WHO clinical stages between genders.

Comparison of QOL between our study population and China’s general population

As shown in Table 2, scores of physiological psychological social relation and environmental domains, and total score of QOL were 14.99±2.25, 14.25±2.12, 13.22±2.37, 13.31±1.99, and 15.99±1.99 respectively. Except the QOL score in environmental domain and the total QOL score, the scores in other three domains had no significant difference with those of China’s general people (*P*<0.05).

**Table 2:** QOL's comparison between HIV/AIDS patients in Zhejiang and domestic general population($\bar{\text{x}}$ ± s).

Domain	HIV/AIDS	Domestic norm	t value	*P value*
Physiological	14.99±2.25	15.10±2.30	-0.38	>0.05
Psychological	14.25±2.12	13.89±1.89	1.73	>0.05
Social relation	13.22±2.37	13.93±2.06	-2.83	>0.05
Environmental	13.31±1.99	12.14±2.08	4.95	<0.05
Total score of QOL	15.99±1.99	13.38±2.91	6.19	<0.05

### Quality of Life

PLWHA in Taizhou had a mean QOL score of 14.99±2. 25 in physical domain, 14.25±2.12 in psychological domain, 13.22±2.37 in social domain, and 13.31±1.99 in environmental domain, with the score in physical domain ranking the highest and that in social domain ranking the lowest. There was statistically significant difference in the environmental domain score between three groups, with homo males having the highest score of 13.81 ±2.01.Subjects aged<30 years had better scores in all domains (*P*< 0.01). Those with higher education level had better scores in physical and environmental domains (*P*<0.05).Those who were single had significantly better scores in all domains than those in other marital status (*P*<0.05).There were statistical differences in scores in physical, social, and environmental domains between farmers and not farmers, with farmers receiving lower scores than not farmers (*P*<0.05). Those infected with HIV through homosexual transmission had significantly higher scores in environmental domain than those infected through other routes (*P* = 0.005). There were significant differences in scores among four CD4 count groups, with the scores in social and environment domains increasing with the number of CD4 counts(*P* <0.05). Those who were on ART had relatively higher scores in all domains than those without ART (*P* <0.05); those at lower WHO clinical stage and with good ART adherence were likely to have better scores than those at higher WHO clinical stage and with poor adherence of ART (*P*<0.05). There was no significant difference in scores in all domains.among different treatment duration groups. Comparison of the mean scores of QOL according to socio-demographic, clinical and disease-related characteristics is presented in Table 3.

Specially, the subgroup analysis showed that homo males who are young, single, not on ART, and with no leakage of drugs, had better scores in physical domain; The analysis of the disaggregated data (subgroups: hetero male, homo male and female) is presented in supporting Information (S1 Table, S2 Table, and S3 Table).

**Table 3:** Comparison of mean scores of quality of life according to socio-demographic, clinical and disease-related characteristics.

Variables	Physiological domain	Psychological domain	Social relation domain	Environmental domain	Total score of QOL
**Gender**					
Hetero male (n = 175)	14.88±2.28	14.14±2.01	13.14±2.32	13.18±2.05	16.01±1.88
Homo male (n = 117)	15.08±2.44	14.48±2.35	13.47±2.53	13.81±2.01	15.93±2.28
Female (n = 111)	15.07±1.99	14.20±2.04	13.11±2.31	13.00±1.83	16.01±1.82
P value	0.692	0.371	0.413	**0.040** *	0.941
**Age(yrs)**					
<30(n = 79)	15.92±1.79	14.99±1.89	14.08±1.94	14.07±1.77	14.77±1.61
30∼(n = 144)	15.08±2.30	14.06±2.39	13.01±2.56	13.17±2.03	13.83±2.03
40∼(n = 99)	14.75±2.26	13.99±1.94	13.01±2.39	12.93±1.98	13.67±1.86
50∼(n = 52)	14.74±2.33	14.42±1.98	13.28±2.55	13.49±2.21	13.98±1.98
≥60(n = 29)	13.34±1.84	13.82±1.70	12.6±1.58	12.95±1.61	13.18±1.42
*P* value	**0.000** **	**0.007** **	**0.006** **	**0.002** **	**0.000** **
**ethnic**					
Han(n = 382)	15.01±2.26	14.26±2.14	13.28±2.37	13.36±1.99	13.98±1.92
Minority(n = 21)	14.69±2.03	14.22±1.76	12.25±2.41	12.50±1.96	13.42±1.73
*P* value	0.532	0.943	0.055	0.056	0.193
**Educational status**					
None(n = 20)	14.34±2.18	14.20±1.47	12.40±1.94	12.70±1.56	13.41±1.54
Primary(n = 91)	14.52±2.04	13.98±1.99	12.90±2.41	12.87±2.04	13.57±1.79
Secondary(n = 183)	15.11±2.43	14.20±2.03	13.28±2.30	13.21±1.89	13.95±1.82
Tertiary and above(n = 109)	15.31±2.39	14.59±2.43	13.57±2.51	13.97±2.06	14.36±2.14
*P* value	**0.041** *	0.215	0.090	**0.000** **	**0.016** *
**Marital status**					
Single(n = 113)	15.69±2.21	14.61±2.31	13.55±2.50	13.9±1.96	14.43±2.02
Married(n = 228)	14.8±2.24	14.25±2.02	13.27±2.24	13.22±1.90	13.89±1.79
Divorced or widowed(n = 62)	14.43±2.12	13.61±2.00	12.47±2.53	12.57±2.15	13.27±1.95
*P* value	**0.000** **	**0.012** *	**0.015** *	**0.000** **	**0.000** **
**Occupation**					
Farmer(n = 139)	14.67±2.09	14.03±1.77	12.85±2.34	12.99±1.83	13.64±1.69
Not farmer(n = 264)	15.16±2.32	14.37±2.28	13.42±2.38	13.48±2.07	14.11±2.00
*P* value	**0.038** *	0.121	**0.023** *	**0.020** *	**0.018** *
**Area**					
inner province(n = 314)	15.00±2.23	14.30±2.05	13.29±2.35	13.38±1.99	13.99±1.87
other province(n = 89)	14.97±2.34	14.08±2.35	13.00±2.49	13.07±2.01	13.78±2.05
*P* value	0.922	0.386	0.323	0.190	0.358
**Transmission routes**					
heterosexual transmission(n = 276)	14.93±2.19	14.13±2.03	13.09±2.33	13.09±1.96	13.81±1.84
homosexual transmission(n = 117)	15.08±2.44	14.48±2.35	13.47±2.53	13.81±2.01	14.21±2.10
other transmission(n = 10)	15.77±1.43	15.00±1.67	14.13±1.43	13.50±2.01	14.60±1.23
*P* value	0.448	0.170	0.163	**0.005** **	0.088
**Reported STD symptom (n = 396)**					
Yes(n = 75)	15.09±2.24	14.31±1.84	13.08±2.23	13.31±1.87	13.95±1.76
No(n = 248)	15.05±2.19	14.28±2.24	13.30±2.40	13.36±1.99	13.99±1.92
NA(n = 73)	14.66±2.48	14.01±1.99	12.99±2.48	13.05±2.19	13.68±2.06
*P* value	0.394	0.595	0.560	0.522	0.456
**CD4 cell count(cells/mm** ^**3**^ **) (n = 397)**					
≤200(n = 64)	14.57±2.10	13.88±2.03	12.75±2.46	12.88±2.05	13.52±1.91
201∼349(n = 105)	14.35±2.52	13.87±2.15	12.75±2.47	12.92±2.02	13.47±2.01
350∼499(n = 118)	15.21±2.11	14.40±1.91	13.51±2.36	13.51±1.83	14.16±1.77
≥500(n = 110)	15.67±1.95	14.75±2.28	13.59±2.15	13.72±2.08	14.43±1.85
*P* value	**0.000** **	**0.007** **	**0.011** *	**0.005** **	**0.000** **
**On HAART**					
Yes(n = 286)	15.76±1.71	14.88±1.68	13.78±2.23	13.79±1.70	14.55±1.52
No (n = 117)	14.68±2.37	14.00±2.23	13.00±2.41	13.12±2.08	13.70±2.00
*P* value	**0.000** **	**0.000** **	**0.003** **	**0.002** *	**0.000** **
**WHO clinical stage(n = 285 on HAART)**					
Ⅰ stage(n = 161)	14.97±2.46	14.38±2.38	13.55±2.54	13.63±2.12	14.13±2.14
Ⅱ stage(n = 90)	14.36±2.13	13.48±1.95	12.27±1.96	12.44±1.84	13.14±1.58
Ⅲ and Ⅳ stage(n = 34)	14.12±2.42	13.53±1.86	12.27±2.20	12.50±1.86	13.12±1.82
*P* value	0.052	**0.004** **	**0.000** **	**0.000** **	**0.000** **
**Adherence of HAART(n = 286)**					
Leakage of drug	14.52±2.35	13.87±2.19	12.84±2.35	12.98±2.06	15.65±1.91
No Leakage of drug	16.12±2.12	15.13±2.31	14.39±2.50	14.34±1.88	15.92±2.22
*P* value	**0.001** **	**0.004** **	**0.001** **	**0.001** **	0.469
**Treatment duration(n = 285 on HAART)**					
≤24 months	14.49±2.34	13.90±1.99	12.77±2.43	13.09±2.08	13.5±1.89
25~48 months	14.74±2.41	14.18±2.51	13.05±2.29	13.18±2.08	13.79±2.09
≥49 months	14.95±2.38	13.95±2.32	13.33±2.50	13.09±2.12	13.8±2.12
*P* value	0.416	0.646	0.287	0.946	0.587

Note

*P<0.05 and

**P<0.01

### Results of Multiple Stepwise Regression Analysis

Table 4 shows four distinct models for QOL. For QOL in physical domain, age(*β* = -0.045), the last recorded CD4 count(*β* = 0.002), and ART adherence(*β* = 1.231) are associated factors. For QOL in psychological domain, the last recorded CD4 count (*β* = 0.002) and WHO clinical stage (*β* = -0.437) are associated factors. For QOL in social domain, WHO clinical stage (*β* = -0.704) and ART adherence (*β* = 1.177) are associated factors. For QOL in environmental domain, WHO clinical stage (*β* = -0.538), educational status(*β* = 0.549) and ART adherence(*β* = 1.078).

**Table 4:** Results of multiple stepwise regression analysis for QOL.

Dependent variable	Independent variable	Coefficient	Std. Error	t	*P value*	95.0% Confidence Interval	
	v	s				Lower Bound	Upper Bound
QOL-physical domain	age	-0.045	0.011	-4.013	0.000	-0.067	-0.023
	CD4 count	0.002	0.001	3.477	0.001	0.001	0.004
	adherence of HARRT	1.231	0.446	2.758	0.006	0.352	2.109
QOL-psychological domain	CD4 count	0.002	0.001	3.046	0.003	0.001	0.003
	WHO clinical stage	-0.437	0.189	-2.315	0.021	-0.809	-0.065
QOL-social domain	WHO clinical stage	-0.704	0.203	-3.471	0.001	-1.104	-0.305
	adherence of HARRT	1.177	0.468	2.516	0.012	0.256	2.098
QOL-environmental domain	WHO clinical stage	-0.538	0.173	-3.099	0.002	-0.879	-0.196
	Educational status	0.549	0.148	3.703	0.000	0.257	0.841
	adherence of HARRT	1.078	0.395	2.729	0.007	0.300	1.855

Note: P<0.05

## Discussion

To our knowledge, in China, a few facility-based studies have been conducted to assess the QOL among PLWHA infected through sexual transmission and the impact of ART on them. Our study using the WHOQOL- BREF instrument assessed the QOL among PLWHA in Zhejiang province and attempted to clarify the associated factors. Specially, 97.5% of our subjects contracted HIV/AIDS via sexual contact and 29.0% of those cases were attributed to men having sex with men (MSM) contact, which coincided with the current epidemic characteristics in China (It was reported that by 2013, the percentage of sexually transmitted cases had increased to 90.8%, with the male homosexual transmission rate reaching 21.4%).[25]. Compared with the PLWHA in Henan province infected mainly via contaminated blood products[16] and those in Liaoning province infected mostly through sexual transmission[21], the characteristics of our study population were quite different: 65.51% with regular jobs and 72.46% having received secondary education or above, obviously higher rates than those of PLWHA in Henan and Liaoning.

All these increase the need to assess the QOL among PLWHA in Zhejiang province and to clarify the influential factors involved.

To assess the QOL of PLWHA in Zhejiang province, we compared the QOL scores of our subjects with the results of the study conducted among Chinese general population (1654 participants, 1999). We found that there was no significant difference in QOL in physiological, psychological and social relation domain between our study population and China’s general population, which might be attributed to the fact that 70.97%of subjects had received free ART and 83.88% were with CD4>200. However, significant difference existed in QOL in environmental domain (13.31±1.99 vs.12.14±2.08, p <0.05), which revealed that though the QOL of PLWHA infected via sexual contact has not seriously deteriorated in physiological, psychological and social relation domains, the environment they were living in was quite different from that of sixteen years ago due to Zhejiang position as the most economically advanced area and municipality’s efforts to improve the health care for PLWHA. The survey of QOL was influenced by the subjective consciousness, environment, the way of HIV transmission and the enjoyed national policy of the participants deeply. China has a large area, and it is much different in the environment, culture and local policies in different areas. It is inappropriate to compare with HIV/AIDS patients in other area. Indeed, there is a limitation, the time distance is long with the study in 1999. The local general population should be taken as the control group in future.

In our study, ART was found to be the first strongest factor affecting the QOL of PLWHA. Data analysis showed a significant correlation between acceptance of ART, CD4 levels and the QOL of all domains (P<0.05), which was consistent with the results of Tsevat J’s study [26]: the symptoms of AIDS patients always occurred together with their bad QOL. What’s more, adherence has a very specific meaning in ART. Our study didn’t show significant correlation between the duration of ART therapy and QOL in any domain, unlike those reports showing that the longer ART therapy duration, the better QOL in physical domain[27]. The level of CD4 cells count is an important immunological parameter to evaluate the effect of ART. The Ye P[28] and Riberio RM [29]studies showed that the growth rate of CD4 cells was high during the first year of ART, and afterwards it would undergo some fluctuations due to changes in an individual's immune function, poor adherence, drug side effects and other factors. As shown in this study, the QOL has little to do with the duration of treatment, but much with whether to accept ART treatment, adherence and level of CD4. The Higher CD4 levels, the better ART adherence, the higher QOL of patient's. Higher QOL indicates that the patient will have better ability to cope with his illness and life, Thus, ART therapy should be used as early as possible among PLWHA.

In our study, some significant gender differences were found in QOL in all domains, similar to many other reports showing better QOL among males than among females [18, 30]. And like the results of the studies by Akinboro et al.[31] and others[32], the finding of our study showed that PLWHA ≤30 years had significantly better QOL in all domains, which may be explained by the fact that local young people in Zhejiang province are more open-minded and have higher tolerance to disease.

As mentioned by other reports that better QOL was found among well- educated people [32, 33], we also found that subjects with tertiary or above education reported better QOL in physical and environmental domains (*P*<0.05). The reason may be that people with higher education level have more enlightened attitude towards the disease with the increasing public awareness of HIV. Data from sentinel surveillance systems show that by 2010, basic awareness of HIV among young students (in-school youth) and male migrants (migrant workers) had reached 88.2% and 75.3%[34].

As shown in appendix 2, homo males who are young, single, not on ART, and with no leakage of drugs, had better scores in physical domain. The research conducted by Yuji Feng showed that HIV-infected gay men experienced multiple forms of stigma and discrimination related to sexual orientation, which have been the major barrier for them to seek health services. When developing HIV/AIDS programs, stigma from both outside and inside the MSM community should be taken into consideration[35]. In our study, we also found the barrier to access ART service for young MSM. The one reason was related to stigma and discrimination, the other one was the side effect of the ART drug. However, MSM with no leakage of drugs had higher scores in physical domain. Thus, when providing ART to MSM, much attention should be paid to the elimination of stigma and discrimination for MSM and measures should be taken to enforce the adherence of ART in MSM.

There were several limitations in our study. First, in view of the fact that the WHOQOL-BREF instrument measures QOL within four weeks prior to the interview, the recall bias may influence the information obtained. Second, this study is only a cross-sectional design survey, thus, it is difficult to make any causal conclusion of between socio-demographic and disease-related variables and QOL, and in future, a prospective study need to be conducted to confirm the findings of this study. However, based on the data analysis, we can say factor such as ART adherence have positive impact on the QOL. Third, the QOL comparison was made with the national norm, the results of which were obtained among China’s general population in 1998. It would be more useful if a comparison with non-HIV infected population in Zhejiang province were made. Yet, there isn’t any outcome of QOL among non-HIV infected individuals in Zhejiang province by WHOQOL- BREF instrument. Lastly, we haven’t examined such important variables as stigma, discrimination, and economic and social support. It is known by all that many PLWHA suffer from serious stigmas and discrimination from others [36]. Also, both economic and social supports tend to have beneficial effects on the life of PLWHA.

### Conclusion

In conclusion, our study revealed the present QOL of PLWHA in Zhejiang province. The factors such as being young, single, not farmers, and with higher education level, high level of CD4 count and good ART adherence tend to have positive effects on QOL of PLWHA. We suggest further research should be conducted to gain a more thorough understanding of the factors that influence QOL so that a more appropriate set of comprehensive intervention measures can be developed. Apart from ART, we should pay more attention to the healthcare availability, acquiring new HIV prevention and control information and psychological intervention skills and so on.

## Supporting Information

S1 Table Comparison of mean scores of quality of life according to socio-demographic, clinical and disease-related characteristics (hetero male). (XLSX)

S2 Table Comparison of mean scores of quality of life according to socio-demographic, clinical and disease-related characteristics (homo male). (XLSX)

S3 Table Comparison of mean scores of quality of life according to socio-demographic, clinical and disease-related characteristics (female) (XLSX)
